# Scutellarin inhibits the glioma cell proliferation by downregulating BIRC5 to promote cell apoptosis

**DOI:** 10.1111/jcmm.17788

**Published:** 2023-06-20

**Authors:** Feng Wang, Ma‐ChiCheng Bao, Jing Xu, Lan‐Lan Shi, Rui‐Ze Niu, Ting‐Hua Wang, Jia Liu

**Affiliations:** ^1^ Department of Animal Zoology Kunming Medical University Kunming China; ^2^ Basic Medical School Kunming Medical University Kunming China; ^3^ Laboratory of Neurological Diseases West China Hospital of Sichuan University Chengdu China

**Keywords:** apoptosis, BIRC5, glioma, proliferation, Scutellarin

## Abstract

The expression changes of baculovirus inhibitor of apoptosis repeat‐containing protein5 in brain glioma after administration of Scutellarin was detected. To explore the effort of scutellarin on anti‐glioma by downregulating BIRC5.The effect of scutellarin on tumour growth and animal survival was detected by administering scutellarin to nude mice subcutaneous tumour formation and SD rats in situ tumour formation models. A significantly different gene BIRC5 was found by using the combination of TCGA databases and network pharmacology. And then qPCR was performed to detect the expression of BIRC5 in glioma tissues, cells and normal brain tissues and glial cells. CCK‐8 was used to detect the IC50 of scutellarin on glioma cells. The wound healing assay, flow cytometry and MTT test were used to detect the effect of scutellarin on the apoptosis and proliferation of glioma cells. The expression of BIRC5 in glioma tissues was significantly higher than that in normal brain tissues. Scutellarin can significantly reduce tumour growth and improve animal's survival. After scutellarin was administered, the expression of BIRC5 in U251 cells was significantly reduced. And after same time, apoptosis increased and cell proliferation was inhibited. This original research showed that scutellarin can promote the apoptosis of glioma cells and inhibit the proliferation by downregulating the expression of BIRC5.

## 1 INTRODUCTION

Glioma is considered as the most common primary tumour in the adult brain, with the highest degree of malignancy; among glioma types, glioblastoma is the most invasive and lethal.^1^, ^2^ Although multinational treatment strategies have improved in the past few decades, the prognosis of patients with glioma remains unfavourable.^3^, ^4^ In particular, the median survival time of patients with high‐grade malignant glioma is ≤14 months.^5^, ^6^ And the 5‐year survival rate of glioblastoma patients is <3%.^7^ Therefore, there is an urgency to explore the mechanism of the invasiveness of glioma and to develop new therapeutic strategies, but particularly to find new anticancer drugs.

Scutellarin, also known as Erigeron scutellarin, is a traditional Chinese medicine commonly found in Yunnan, Guizhou, Sichuan and other southwestern provinces. Its chemical name is 4′,5,6‐trihydroxyflavone‐7‐O‐β‐D‐glucuronic acid. Glycosides, which belong to the flavonoid glycoside class, are flavonoids extracted and isolated from the dried whole plant of the Compositae plant (Erigeron scutellarin [Vant] Hand‐Mazz). It is the main component of the clinical medicine scutellarin (>85%). Scutellarin has a variety of pharmacological effects, such as antioxidant,^8^ anti‐inflammatory,^9^ anti‐cancer^10^ and neuroprotection effects.^11^ It has been reported that scutellarin plays a significant role in the treatment of ischemic brain injury diseases, such as cerebral infarction, and the increased production of reactive oxygen species is an important mechanism for the occurrence of ischemic brain diseases. Therefore, the pharmacology action of scutellarin is related to the nature of scavenging reactive oxygen species.^11^ Scutellarin aglycone can improve the neurological dysfunction of middle cerebral artery embolism rats with focal cerebral ischemia in a dose‐dependent manner and reduce the area of cerebral infarction.^12^ In addition, scutellarin has a neuroprotective effect on cerebral ischemia–reperfusion injury,^13^ but there are few reports of this effect in glioma.

Baculovirus inhibitor of apoptosis protein 5 (BIRC5) is a newly discovered member of the inhibitor of apoptosis protein (IAP) family, and its function is the strongest among the inhibitors of apoptosis that have been identified thus far.^14^ BIRC5 is located at 17q25 and is 14.7 kb in length. It has 3 introns and 4 exons, and contains a repetitive sequence of baculovirus IAPS. BIRC5 is abundantly expressed in fetal tissues and various human malignant tumour tissues, but not in normal tissues or well‐differentiated adult tissues.^15^ Previous studies have shown that BIRC5 is highly expressed in glioma and associated with poor prognosis in patients with glioma.^16^, ^17^

In the present study, scutellarin was proven to have significant anti‐glioma effects, and BIRC5 was reported as an oncogene of glioma. Scutellarin exerts an anti‐tumour effect by downregulating the BIRC5 gene. BIRC5 downregulation significantly inhibits the proliferation of glioma cells, in which glioma cell apoptosis is activated.

## 2 MATERIALS AND METHODS

### 2.1 Microarray data

The gene expression profile and clinical‐related data of GBM were downloaded by entering the GDC data in TCGA database; these data which consisted of 330 high‐grade glioma samples and five normal brain tissue samples. The series matrix and platform files were downloaded and saved as .txt files. The downloaded files were processed by the R software package, and the data were also evaluated by the R software package.

### 2.2 Screening of differentially expressed genes

R language software and an annotation package were used to download series matrix and platform files. The ID of the matching probe name was converted into the international standard gene name, and then saved as a txt file. A LIMMA package was used for gene differential expression analysis. The relevant command codes were input into R, and then the LIMMA software package was used to analyse the differentially expressed genes of high‐grade glioma and normal brain tissues in the microarray data set. Samples with fold change (FC) correction values of >6 and *p* < 0.05 were considered to be differentially expressed mRNA genes. The XLS results were saved for subsequent analysis.

### 2.3 Integration of microarray data

The list of differentially expressed genes of the microarray dataset accessed through the LIMMA package was saved as an XLS file. The gplots package was downloaded and the R language software was used to run the instruction code. Subsequent analysis yielded a list of upregulated or downregulated genes in the transcriptomics data.

### 2.4 Identification of scutellarin component targets

The biologically active components of scutellarin were obtained from the traditional Chinese medicine systems pharmacology database and analysis platform (http://lsp.nwu.edu.cn/tcmsp.php). After searching the database and eliminating duplicates, a total of 12 main compounds were obtained. Next, UniProt (http://www.uniprot.org/) was used to confirm gene information, including name and gene ID.

### 2.5 Molecular docking

Based on molecular docking analysis, the important targets and active ingredients obtained from the network pharmacology screening were verified. The corresponding ligands of active ingredients were downloaded from the PubChem and PDB databases. The core protein domain of the target was downloaded, Auto Dock Tools 1.5.7 was then used to dehydrate and hydrogenate the ligands and receptors, and the active site for molecular docking was determined. Finally, the binding energies of proteins and active ingredients were scored and the results were annotated and visualized by PyMOL.

### 2.6 Cell culture

#### 2.6.1 Cell line

The U251 and LN229 glioma cell line (cat. no. 2020.9.12; Kunming Institute of Zoology) was used in in vitro experiments and in vivo tumour formation experiments. All cell lines have been identified. U251 cells grew normally in 89% DMEM/high glucose (HyClone; Cytiva), 10% FBS serum (HyClone; Cytiva) and 1% penicillin–streptomycin solution (HyClone; Cytiva). Following washing once with PBS (HyClone; Cytiva), the cells were digested with 0.25% trypsin (1–2 mL; Gibco) for 2–3 min. The digestion of U251 cells was terminated by DMEM (HyClone; Cytiva) containing 10% FBS. Following centrifugation (300 g, 10 min) and resuspension, the cell suspension was collected, and the cells were placed in a 25 T (3 mL) culture flask at a density of 4 × 10^5^ cells/mL, and placed in an incubator. After 24 h, the supernatant containing non‐adherent cells was removed and fresh medium was added. Once cells had reached close to confluence (80%–90%), the medium was changed every 3–5 days, with a density of ~4 × 10^5^ cells/cm^2^, the cells were passaged three times, and the suspended cells were discarded. Subsequently, purely adherent U251 cells were cultured for further analysis. The growth status of the cultured cells was observed under an inverted microscope (*ECLIPSE Ti, Nikon*).

#### 2.6.2 IC50 determination

The U251 glioma cell line was seeded in a 96‐well plate with 3 × 10^3^ cells per well, and placed in a 37°C, 5% CO_2_ cell incubator overnight. After 24 h, different concentrations of scutellarin were added. Next, 10 μL cell counting kit (CCK‐8) reagent was added to the well, followed by incubation for 2 h in a cell incubator, and absorbance (OD value) was then measured at 450 nm using a microplate reader. In the present study, the drug group was set as A, the control group (DMSO group) as B and the blank control group as C, with a cell viability rate of (A‐C)/(B‐C) × 100%.

#### 2.6.3 Clone formation

The U251 cells were digested with 0.25% trypsin when cell viability was good, and the cells were counted on a cell counting board. A total of 500 cells were added to each well in the 6‐well plate and placed in the cell incubator at 37°C overnight. The state of the cells was observed under an inverted microscope the next day. After all the cells had adhered to the wall, the medium containing dead cells was abandoned, the fresh 10% FBS complete medium and scutellarin were added to the corresponding concentration, and the 6‐well plate was put back into the incubator for culture. The state of the cells was observed every 2 days and the culture medium was changed every 4 days. When the cell community exceeded 50 cells, a picture was captured under the microscope and it was recorded; it was then washed with PBS that had been warmed up in advance, and the remaining PBS was dried in the hole with a pipette. Cells were then fixed for 20 min with 4% paraformaldehyde preheated by 1 mL in each hole, and an appropriate amount of PBS was added to clean the cells three times. A total of 200 μL 0.5% crystal violet dye solution was added to each hole to dye for 20 min; the holes were then washed with pure water three times and covered using an orifice plate cover and left to dry overnight. In addition, the cell communities of >30 pixels in each orifice plate were calculated using ImageJ software (National Institutes of Health) and were statistically analysed compared with the Control group.

#### 2.6.4 Wound healing assay

U251 cells were digested and counted. Next, 1 × 10^6^ cells were seeded to each well of a 6‐well plate and incubated overnight in an incubator at a constant temperature of 37°C. Using the 10‐μL pipette, the monolayer cells were scratched with the word ‘#’ on the orifice plate, the complete medium containing 10% FBS was added, the 6‐well plate was gently shaken and the medium discarded. The steps above were repeated three times to remove the cells caused by scratches. Fresh and complete medium containing scutellarin was added, and the culture flask was placed in an incubator with 5% CO_2_ at a constant temperature of 37°C. The images of eight visual fields in each group at 0, 24 and 48 h were taken at the same position using an inverted microscope. In addition, the area of each scratch at 0, 24 and 48 h were measured using ImageJ software, and compared with the Normal and Control groups.

#### 2.6.5 Cell apoptosis detection by flow cytometry

U251 cells were seeded in a 6‐well plate with 10^6^ cells per well, and incubated in a cell incubator with 5% CO_2_ 37°C, overnight. After 24 h, different concentrations of scutellarin were added, and the supernatant was discarded after 48 h. The solution was washed three times with 0.01 M PBS, for 5 min each time. The cells were digested with 0.25% trypsin. After 2 min of digestion, DMEM complete medium containing 10% fetal bovine serum was added to stop the digestion, centrifuged (300 g, 10 min), and the supernatant was discarded. Next, 0.01 M PBS was added to wash three times. Next, 100 μL binding buffer was added to each tube to create a cell suspension, and the treated cells were single‐ and double‐stained with AV‐FITC and PI, respectively, followed by incubation for 15 min at room temperature in the dark. After incubation, 400 μL binding buffer was added to each tube. Flow cytometry was used to determine cell apoptosis, and the positive results of AV‐FITC and PI were statistically analysed.

#### 2.6.6 Proliferation detection by CCK‐8

U251 cells were seeded in a 96‐well plate with 3000 cells per well, and placed in a cell incubator with 5% CO_2_ at 37°C overnight. After 24 h, different concentrations of scutellarin were added. After 48 h of treatment, 10 μL CCK‐8 solution was added per well and incubated for 2 h in the incubator. A microplate reader was used to measure the absorbance of each well at a wavelength of 450 nm. The OD value of each hole was subtracted from the OD value of the blank control group, and the average value ± SD value of each replicate hole was the final result.

#### 2.6.7 Reverse transcription‐quantitative PCR (RT‐qPCR)

U251 cells in the logarithmic growth phase were inoculated in 10% FBS DMEM complete medium. The cell density was then adjusted to 1 × 10^6^ cells/mL, followed by additional inoculation into a 6‐well plate with 2 mL 10% FBS DMEM complete medium per well. The cells were treated with scutellarin at a concentration of 338.4 μM, and 3 replicate wells were used in each group. After 48 h of culture, the total RNA of each group of cells was extracted using TRIzol™ reagent (Hangzhou). After the concentration and purity of the RNA were detected using a microplate reader, the RNA was reverse‐transcribed to synthesize cDNA and then amplified by PCR. Reaction system (total volume, 20 μL): 1 μL cDNA template, 10 μL Master Mix, 0.6 μL upstream and downstream primers and 8.8 of diethylpyrocarbonate water. Amplification conditions: 40 cycles of 95°C pre‐denaturation for 30 s; 95°C denaturation for 5 s, 50°C annealing for 30 s and 72°C extension for 20 s. Using β‐actin as an internal reference, the 2^−ΔΔCt^ method was used to calculate the expression level of the BIRC5 and COL3A1 gene. The experiment was repeated three times. The primer sequence and product length are shown in Table 1.

**TABLE 1:** Primer sequence and product length.

Gene name	Upstream primer sequence (5′‐3′)	Downstream primer sequence (5′‐3′)	Product length (bp)
β‐Actin	CTCGCCTTTGCCGATCC	GAATCCTTCTAGCCCATGCC	203
BIRC5	CCTGGCTCCTCTACTGTT	CTCTATTCTGTCTCCTCATCC	157
COL3A1	CTACGGCAATCCTGAACTT	GCAACCATCCTCCAGAAC

#### 2.6.8 Rat orthotopic tumour model

The animal study was reviewed and approved by the Medical Ethics Committee of Kunming Medical University. A total of 60 female SD rats weighing 180–220 g were selected and fasted for 24 h pre‐operatively, except for an appropriate amount of water. Rats were anaesthetised by 2% isoflurane inhalation, and all rats were inoculated using the stereotactic method. The incision and drilling location was determined according to the Barker method, and the right caudate nucleus was selected as the target on the rat brain stereotaxic instrument. The coordinates were as follows: 1.0 mm behind the midpoint of the bregma, and the sagittal suture opens on the right side. 3.5 mm, 5.0 mm subdural. The hair at the top of the head was cut off, followed by disinfection with iodine and alcohol; a surgical drape was then spread. The skin longitudinally backward from the intersection of the mid‐sagittal plane of the head was cut to expose the skull marks and locate according to the aforementioned coordinate points. A dental drill was used to drill a hole on the skull to develop an aperture of 1.2 mm. A 10‐μL micro syringe needle was used to pierce the dura mater. Under stereotactic guidance, the needle was vertically and slowly extended to the subdural 6.0 mm, then 1.0 mm was delayed at the subdural 5.0 mm target. Next, 10 μL U251 cell suspension (1 × 10^7^ cell/mL) was slowly injected into the target area at 1 μL/min, and the needle was retained for 5 min to fully deposit the cells and finally suture the scalp. Finally, all rats were euthanized by excessive isoflurane inhalation. The standard of death was cardiac arrest.

#### 2.6.9 Nude mouse subcutaneous tumour model

The animal study was reviewed and approved by the Medical Ethics Committee of Kunming Medical University. A total of 60 female nude mice weighing 18–22 g were selected and fasted for 24 h pre‐operatively, except for an appropriate amount of water. Next, they were anaesthetised with 2% isoflurane inhalation. Under aseptic conditions, 0.2 mL cell suspension was absorbed using a 1‐mL syringe, and 0.2 mL (5 × 10^7^cell/mL) U251 cell suspension was injected subcutaneously into the axillary skin of the right forelimb of nude mice. The axillary skin of the right forelimb was pulled up and pierced subcutaneously, and the tumour cell suspension was injected quickly. At moment, a round mass full of tension was formed under the skin. Cotton swabs were used to press the needle into it and pull it out quickly to prevent tumour cells from overflowing. Finally, all nude mice were euthanized by excessive isoflurane inhalation. The standard of death was cardiac arrest.

#### 2.6.10 Scutellarin administration

A total of 60 rats and 60 nude mice were randomly divided into 4 groups, namely the DMSO negative control group, temozolomide positive control group, scutellarin low‐dose treatment group (20 mg/kg) and scutellarin high‐dose treatment group (50 mg/kg),^18^ 15 in each group. The intraperitoneal injection was administered once a day, and the body weight, water and diet and the tumour volume‐related indicators of rats and nude mice were monitored.

#### 2.6.11 PET/CT scan

Before the PET/CT test, the rats and nude mice were fasted for 8 h and were water‐free for 4 h. Following isoflurane inhalation anaesthesia, each rat and nude mouse was injected intraperitoneally with 18F‐FDG 4.2 MBq/kg and then fixed in the prone position on the micro‐positron emission tomography (PET)/computed tomography (CT) scanning bed (Discovery 690/Elite; GE Healthcare), using AW Volume Share 5 software (France) to collect static images for 20 min. The scanning parameters were as follows: Voltage, 120 kV; current, 260 μA; pitch, 0.561; rotation speed, 0.5 s/cycle; layer thickness, 3.75 mm; interval, 3.75 mm; matrix 512 × 512; FOV, 50 × 50 cm; subsequent PET scan; parameters for each rat or a nude mouse scans two beds, each bed is collected for 2.5 min. CT was used for attenuation correction and iterative reconstruction to obtain 47 frames of PET cross‐sectional images. The CT and PET image data were transferred to the AW Volume Share 5 workstation, and the coronal, sagittal, cross‐sectional and three‐dimensional CT and PET scans and their fusion images were obtained. Two PET/CT rapporteurs read the PET/CT images using double‐blind method, the tumour and normal tissue ROI was delineated, and the SUVmax was measured.

#### 2.6.12 Sample collection

Six freshly resected glioma and paracancerous tissues were collected from patients in the First affiliated Hospital of Kunming Medical University. The tumour tissues were divided into two parts and numbered; one was stored in 4% paraformaldehyde for Haematoxylin–eosin (HE) and immunohistochemical staining and the other was immediately stored in a liquid nitrogen tank for RT‐qPCR. Informed consent for sample collection was obtained from the patients prior to participation in the study.

#### 2.6.13 Haematoxylin–eosin (H&E) staining

The glioma tissue was dehydrated by automatic dehydrator, made transparent with xylene, soaked in wax and embedded in paraffin. Tissues were then sectioned at 4 μm. The slices were then dewaxed, hydrated, stained with H&E, dehydrated, made transparent with xylene and sealed. Morphological changes were observed under a light microscope, and images were captured.

#### 2.6.14 Immunohistochemical staining

Tissues were sectioned and embedded in paraffin, and ~ 150 μL normal non‐specific goat serum sealing solution was added and incubated at room temperature for 1 h. Primary antibodies CD34 (1:500, Shanghai) and GFAP (1:300, Shanghai) were then added (200 μL per tissue slide), followed by incubation at room temperature for 1 h. Subsequent incubation was performed in a refrigerator at 4°C for 14 h overnight, and at room temperature for 1 h the next day. PBS was then used for rinsing every 5 min for a total of six times. Subsequently, ~150 μL fluorescent secondary antibody was added to each tissue slide, followed by rinsing with PBS every 5 min for a total of five times after incubation at room temperature for 2 h. Finally, the tablets were sealed using a water‐based sealing agent, and the images were observed and collected under the fluorescence microscope.

#### 2.6.15 Statistical analysis

All experimental data of this original research were statistically analysed using SPSS v.21 software (IBM Corp.). Comparisons between two groups were made using an independent sample *t*‐test, and comparisons between three or more groups were made using one‐way anova. Data are expressed as the mean ± SD. *p* < 0.05 was considered to indicate a statistically significant difference.

## 3 RESULTS

### 3.1 Effects of scutellarin on body weight and survival time of tumour‐bearing rats in situ

**FIGURE 1:**
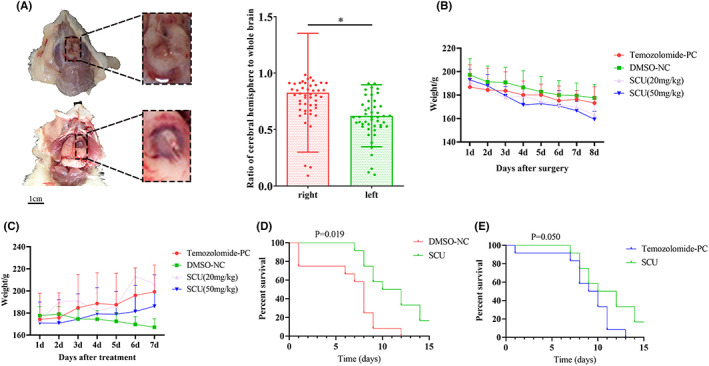
The effect of scutellarin on tumour‐bearing rats in situ. (A) Brain anatomy and PET‐CT in rats with glioma tumour formation in situ (*n* = 48). (B) The body weight change of the orthotopic tumour model rat 7 days after surgery (*n* = 15). (C) Changes of body weight in orthotopic tumour model rats 7 days after administration (*n* = 15). (D) Survival analysis of high‐dose scutellarin treatment group and negative control group in orthotopic tumour model rats after treatment (*n* = 15). (E) Survival analysis of high‐dose scutellarin treatment group and temozolomide group in orthotopic tumour model rats after treatment (*n* = 15). Data are expressed as mean ± standard deviation. *p < 0.05.

First, SD rats were used to establish an orthotopic tumour model. In order to evaluate whether the glioma model was successful, rats were randomly selected for brain dissection at 7 days to see if the tumour had formed, and the remaining rats then underwent a PET‐CT scan to confirm the formation of tumours in the brain. The results showed that all rats had tumours in the brain at 7 days (Figure 1A). Next, all the rats were treated with scutellarin. The weight of the rats was found to have significantly decreased 7 days post‐operatively (Figure 1B), which was consistent with the clinical primary glioma patients.^19^ But interestingly, during the treatment phase after tumour formation, the weight of the rats in the DMSO group continued to decrease (Figure 1C), and the rats in the DMSO group died on the first day of treatment with scutellarin (Figure 1D). This showed that in the early stage of glioma growth, the tumour consumes a lot of nutrition and leads to the weight loss of rats. After treatment with scutellarin, the growth of glioma is inhibited and the body weight of rats gradually recovers. No significant difference was observed between the scutellarin and other groups, which showed that scutellarin has no other obvious side effects on rats. Next, the survival of all rats was evaluated, and it was shown that scutellarin effectively increased the survival rate of rats compared with the DMSO negative control (Figure 1D); then, as compared with the temozolomide group, a certain difference in the survival improvement of rats was observed following treatment with scutellarin (Figure 1E). In summary, scutellarin can improve the survival of rats with glioma, and has a certain anti‐glioma effect.

### 3.2 Effect of scutellarin on body weight, tumour volume and survival time of subcutaneous tumour‐bearing nude mice

**FIGURE 2:**
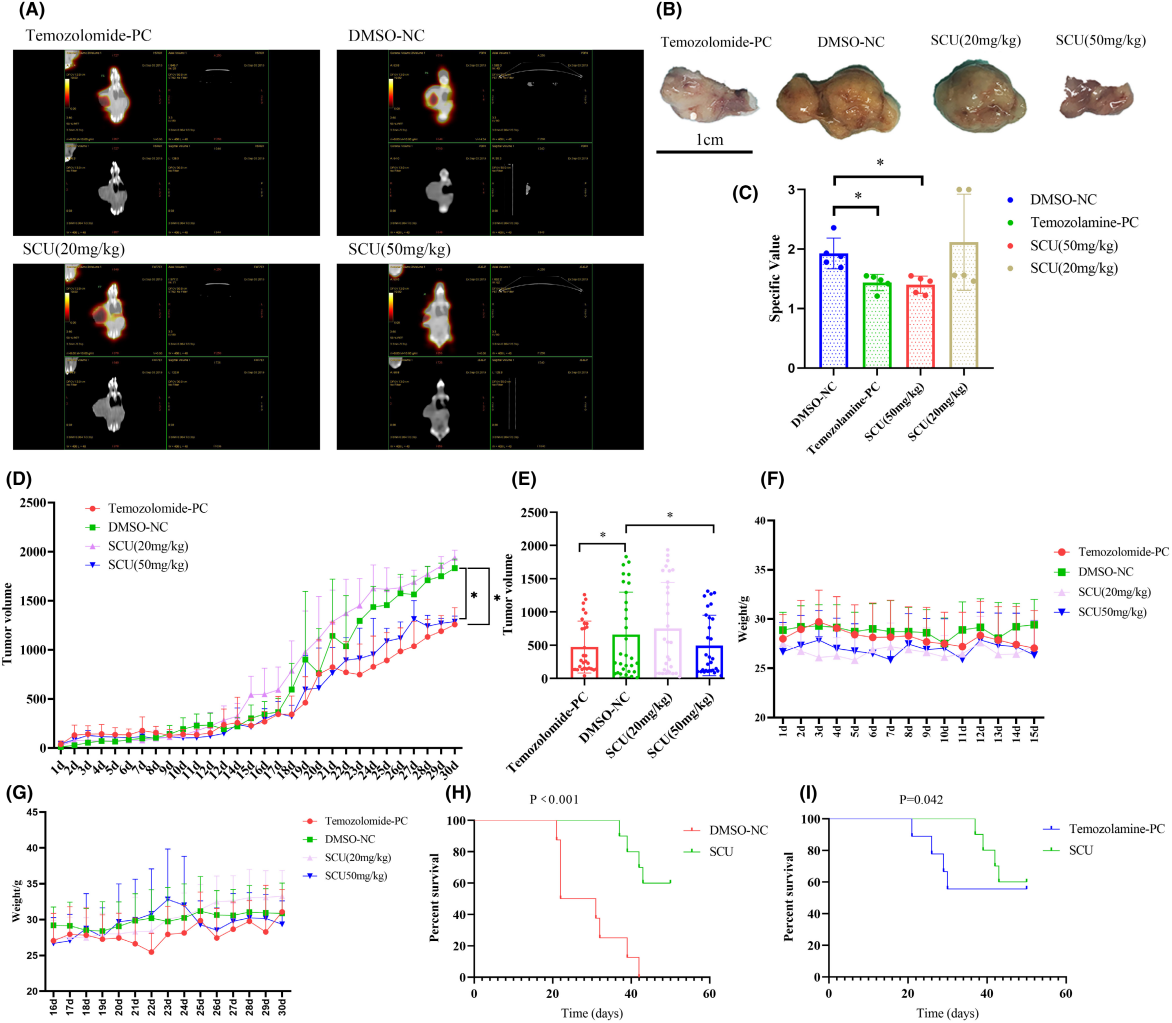
The effect of scutellarin on subcutaneous tumour‐bearing nude mice. (A) PET‐CT scan of nude mice with glioma subcutaneous tumours (*n* = 5). (B) Anatomy of tumour‐forming nude mice and metabolic values of each group (*n* = 5). (C) Changes in tumour volume of nude mice in each group (*n* = 16). (D) Comparison of tumour volume changes of nude mice in each group, and the data are expressed as mean ± standard deviation (*n* = 16). (E) The body weight change of nude mice of subcutaneous tumour model 15 days after administration (*n* = 16). (F) The weight change of nude mice in subcutaneous tumour model 16–30 days after administration (*n* = 16). (G) Survival analysis of the subcutaneous tumour model in nude mice after surgery in the high‐dose scutellarin treatment group and the DMSO group (*n* = 16). (H) Survival analysis of the subcutaneous tumour model in nude mice after surgery in the high‐dose scutellarin treatment group and the temozolomide group (*n* = 16). Data are expressed as mean ± standard deviation. *p < 0.05.

In order to further study about the anti‐cancer effect of scutellarin in vivo, nude mice were used to establish a subcutaneous tumour model. And in order to evaluate the success of the nude mouse model, one nude mice were randomly selected from each group for dissection. The results showed that the subcutaneous mass was glioma (Figure 2B). The nude mice with successful tumour model construction were grouped and administered. After 30 days of administration, all nude mice underwent a PET‐CT scan. The PET‐CT results showed that the nude mouse tumour volume was significantly reduced by high‐dose scutellarin treatment (Figure 2A). At the same time, the metabolic value was significantly lower than that of the DMSO negative control group (Figure 2C). The tumour volume of nude mice was monitored daily. The results showed that the tumour volume of scutellarin‐ and temozolomide‐treated nude mice was significantly smaller than that of DMSO group mice during the first month of treatment (Figure 2D,E). The body weight of nude mice over the past month was analysed and the results showed that the body weight of the nude mice was slightly decreased in the first 15 days (Figure 2F). Of note, the body weight of the DMSO group was maintained at a certain level in the last 15 days, while the body weight of other groups increased to a certain extent (Figure 2G). This result indicated that the drug had an inhibitory effect on tumour growth and regulated the homeostasis of nude mice. In the analysis on the survival time of nude mice, it was found that scutellarin significantly improved the survival of nude mice (Figure 2H,I). The above results indicated that scutellarin can effectively inhibit glioma growth. At the same time, it can significantly improve the survival rate of nude mice, and regulate their body homeostasis.

### 3.3 Effects of scutellarin on U251 cell proliferation

**FIGURE 3:**
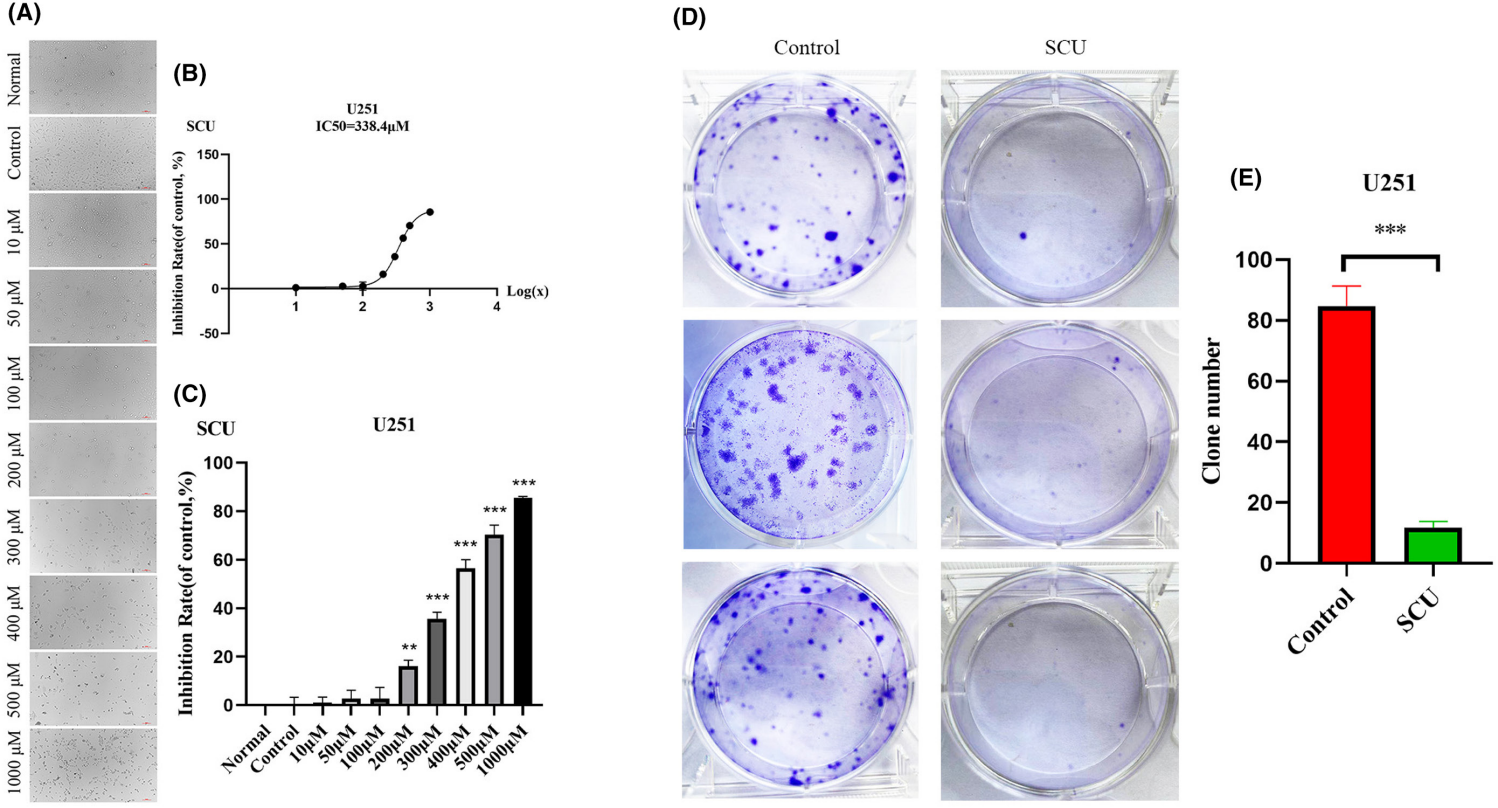
Effect of scutellarin on the proliferation of U251 cells. (A) Scutellarin was added to U251 cells in gradient. (B) IC50 of scutellarin in U251 cells. (C) The inhibition of scutellarin on the proliferation of U251 cells. (D) The proliferation of U251 cells treated with scutellarin. (E) The number of clones of U251 cells treated with scutellarin. Data are expressed as mean ± standard deviation. ***p < 0.001, **0.001 < *p* < 0.01.

The CCK‐8 assay was used to detect the IC50 of scutellarin on U251 cells. After 48 h of treatment, the IC50 of scutellarin in U251 cells was found to be 338.4 μM (Figure S3A,B) and that in LN229 cells 297.2 μM (Figure 1A). Simultaneously, it could be seen that scutellarin had a dose‐dependent effect on U251 and LN229 cells (Figure 3C and Figure S1B). In order to study whether the dose of IC50 can cause damage to normal glial cells, CCK‐8 was performed to detect the viability of HEB cells at a dose of IC50. The results showed that neither 297.2 nor 338.4 μM could significantly affect the viability of HEB cells (Figure S1C,D), nor did they affect the proliferation rate of HEB cells (Figure S1E). A plate cloning assay was performed to study the effect of scutellarin on U251 cell proliferation. As compared with the Control group, the number of U251 cell clones in the scutellarin group was significantly decreased (Figure 3D,E), which indicated that scutellarin could inhibit the U251 cell proliferation.

### 3.4 Effects of scutellarin on U251 cell migration

A scratch experiment was carried out in order to study whether scutellarin can affect the metastasis of glioma. Following drug intervention for 24 and 48 h, it was found that scutellarin significantly inhibited the migration of U251 cells, as compared with the Normal and Control groups (Figure S2A–C). The results revealed that scutellarin could effectively inhibit the proliferation and migration of U251 cells.

### 3.5 Identification of differentially expressed genes and scutellarin targets in glioma

**FIGURE 4:**
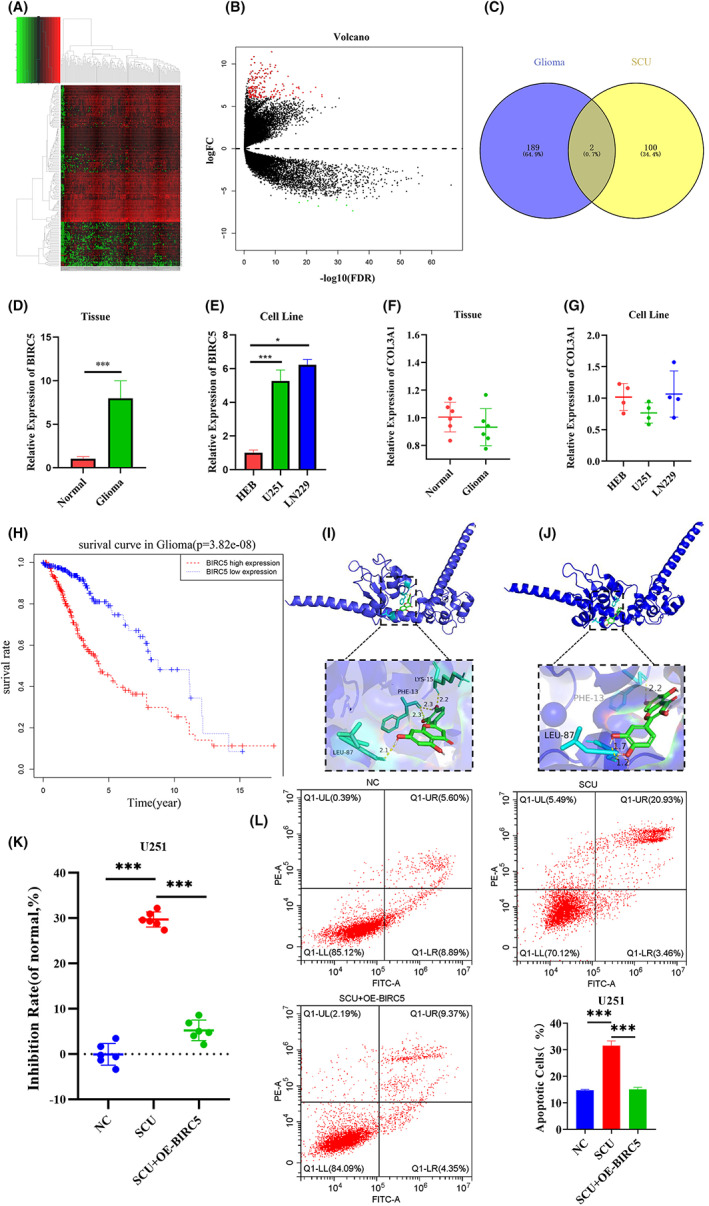
Identification of differential genes and scutellarin targets for glioma. (A) Hierarchical clustering heat map of differentially expressed genes were screened based on log Fold Change>6.0 and corrected *p* value <0.05. (B) Volcano map of differential expression genes, red means up‐regulation and green means downregulation. (C) Venn diagram, blue indicates the target of glioma, yellow indicates the target of scutellarin and grey indicates the intersection of the two. (D) The relative expression level of BIRC5 in glioma tissues (*n* = 6). (E) The relative expression level of BIRC5 in cell lines (*n* = 4). (F) The relative expression level of COL3A1 in glioma tissues (*n* = 6). (G) The relative expression level of COL3A1 in cell lines (*n* = 4). (H) The overall survival rate of glioma patients was evaluated according to the expression level of BIRC5. (I) Molecular docking diagrams of luteolin and BIRC5. (J) Molecular docking diagrams of quercetin and BIRC5. (K) Inhibition rate of overexpressing BIRC5 in scutellarin treated glioma cells (*n* = 6). (L) Apoptotic cells of overexpressing BIRC5 in scutellarin treated glioma cells (*n* = 3). Data are expressed as mean ± standard deviation. ***p < 0.001, **0.001 < *p* < 0.01, * *p* < 0.05.

The transcriptomics and clinical data of glioma were downloaded from TCGA database. A total of 191 differentially expressed mRNAs were obtained using the edgeR package according to the screening of the dataset (corrected *p* < 0.05, logFC>6), (Table SI), including 185 upregulated and 6 downregulated genes (Figure 4A,B). In order to study the mechanism of scutellarin against glioma, the target of scutellarin was queried using The Traditional Chinese Medicine Systems Pharmacology Database and Analysis Platform, and then intersected with DEGs in glioma to obtain two key targets, BIRC5 and collagen, type III, alpha 1 (COL3A1) (Figure 4C). Clinical glioma tissues were therefore collected and identified using H&E and immunohistochemical staining in order to verify whether the tissues were gliomas. The results showed that all six cases were gliomas (Figure S3). RT‐qPCR was performed to detect the expression of BIRC5 and COL3A1 in glioma tissues and cells. It was found that BIRC5 had an abnormally high expression in glioma tissues and cells (Figure 4D,E), while no significant change was observed in the expression of COL3A1 (Figure 4F,G). In addition, the clinical follow‐up data of glioma patients from TCGA database was analysed, and it was found that the 5‐year survival rate of patients with BIRC5 upregulation was significantly lower than that of patients with BIRC5 downregulation (Figure 4H). Among them, BIRC5 was highly expressed in renal cell carcinoma,^20^ pancreatic cancer,^21^ colorectal cancer^22^ and breast cancer^23^ and inhibited tumour cell apoptosis. The mode of action between BIRC5 and quercetin or luteolin in scutellarin was studied by molecular docking. The results showed that BIRC5 can bind to the two pharmacological components of scutellarin, and the binding mode and binding site between them is shown in Figure 4I,J. In addition, their binding energies were found to all be <−5, suggesting that the binding activity between molecules is good. In order to further prove that scutellarin can target and regulate BIRC5, BIRC5 was overexpressed in scutellarin treated glioma cells. The results showed that SCU + OE‐BIRC5 could greatly reduce the inhibition of cell proliferation induced by scutellarin (Figure 4K). At the same time, it also reversed the apoptosis of glioma cells induced by scutellarin (Figure 4L). Therefore, BIRC5 is a reliable target of scutellarin. In conclusion, scutellarin may play an anti‐glioma role by regulating BIRC5 and affecting apoptosis.

### 3.6 Effects of scutellarin on the apoptosis and proliferation of U251 cells following BIRC5 downregulation

**FIGURE 5:**
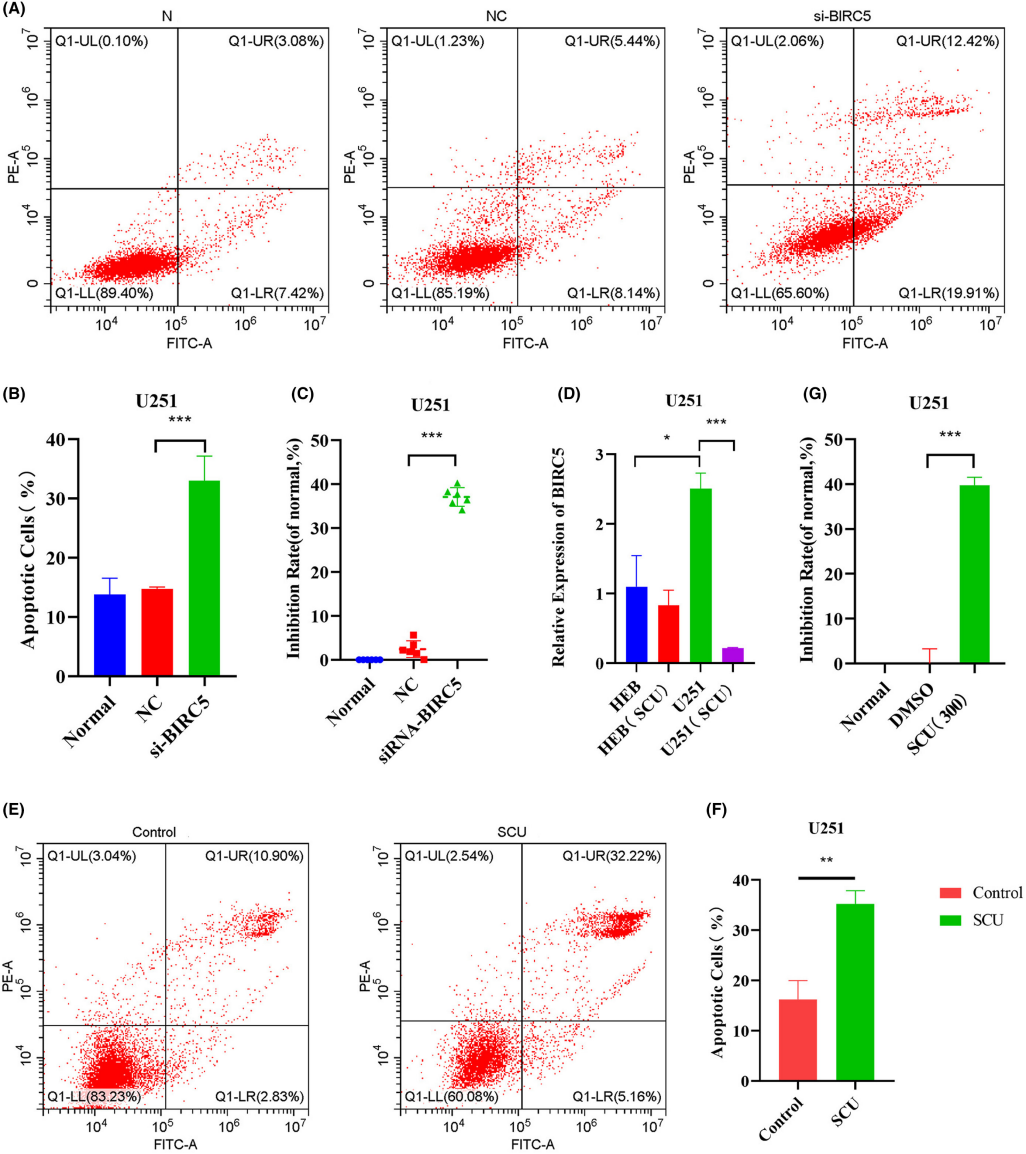
The effect of scutellarin on apoptosis and proliferation of U251 cells after downregulation of BIRC5. (A) Apoptosis map shows the apoptosis of U251 cells after BIRC5 interference, from left to right are Normal, NC and siRNA‐BIRC5. (B) Apoptotic numbers of U251 cells after IRC5 interference (*n* = 3). (C) Inhibition rate of BIRC5 interference on U251 cells (*n* = 3). (D) Relative expression of BIRC5 in HEB and U251 cells treated with scutellarin for 48 h (*n* = 4). (E) Apoptotic map showed that the apoptosis of U251 cells treated with scutellarin for 48 h, from left to right are Control and SCU (*n* = 6). (F) Apoptosis of U251 cells treated with scutellarin for 48 h (*n* = 3) (G) Inhibition rate of U251 cells treated with scutellarin for 48 h (*n* = 6). Data are expressed as mean ± standard deviation. ***p < 0.001, **0.001 < *p* < 0.01, *p < 0.05.

BIRC5 is an apoptosis‐suppressing gene, which plays a role in inhibiting apoptosis in tumours. For the sake of exploring whether BIRC5 downregulation can promote glioma cell apoptosis, the interference fragments of BIRC5 were screened (Figure S4A,B). The apoptosis of U251 cells was analysed by flow cytometry following the addition of si‐BIRC5. The results showed that apoptosis increased significantly following the addition of si‐BIRC5 (Figure 5A,B), and cell proliferation was significantly inhibited (Figure S4C and Figure 5C). In order to make sure whether scutellarin promotes the apoptosis and inhibits the proliferation of glioma cells by downregulating BIRC5, scutellarin was added to U251 and LN229 cells, RT‐qPCR was used to detect the expression of the BIRC5 gene in U251 and LN229 cells after 48 h. The expression of BIRC5 in U251 and LN229 cells following scutellarin administration was significantly lower than that in untreated U251 and LN229 cells, and significantly lower than that in HEB cells (Figure 5D and Figure S4D). Of note, scutellarin can only slightly reduce the expression of BIRC5 in normal HEB glial cells (Figure 5D). These results indicated that scutellarin can significantly downregulate BIRC5 in glioma cells. In addition, flow cytometry was used to analyse the apoptosis of U251 cells following the addition of scutellarin for 48 h. It was found that scutellarin significantly increased U251 cell apoptosis (Figure 5E,F). At the same time, CCK‐8 assay showed that scutellarin significantly inhibited U251 and LN229 cell proliferation (Figure 5G and Figure S4E), but the same dose of scutellarin did not inhibit HEB cell proliferation (Figure S4F). The above results suggested that scutellarin can downregulate BIRC5 to promote glioma cell apoptosis and inhibit their proliferation.

## 4 DISCUSSION

Glioma accounts for 80% of all primary malignant tumours of the central nervous system.^24^, ^25^ At present, the standard treatment for glioma is maximal surgical resection, supplemented with radiotherapy and chemotherapy. However, post‐treatment glioma recurrence occurs frequently, and the prognosis is unfavourable.^26^ Therefore, identifying the mechanism of the occurrence and development of glioma is urgent, in order to explore new therapeutic drugs and methods.

Scutellarin is a flavonoid glycoside compound with a variety of pharmacological activities and has been clinically used to treat various cardiovascular and cerebrovascular diseases.^27^ In recent studies, scutellarin has been reported to have marked anti‐tumour effects. Deng et al.^28^ found that scutellarin inhibits the proliferation and migration of human renal cancer cells by upregulating PTEN. Zhu et al.^29^ reported that scutellarin inhibits the metastasis and angiogenesis of human colorectal cancer by targeting ephrinb2. In addition, Hou et al.^30^ found that scutellarin can inhibit the proliferation, invasion and tumorigenicity of human breast cancer cells by regulating the HIPPO‐YAP signalling pathway. Consistent with previous reports, our results showed that BIRC5 upregulation can promote the proliferation and migration of glioma cells, and scutellarin can significantly inhibit or even block these. The present study explored the effect of scutellarin on the survival and status of tumour‐forming rats in situ, and nude mice with subcutaneous tumours. The results showed that scutellarin can improve the living conditions of rats and nude mice and regulate homeostasis. The most important thing was that it can effectively inhibit tumour growth. In addition, we also studied the effects of scutellarin on the proliferation and migration of the U251human glioma cell line. The results showed that scutellarin inhibited the proliferation and migration of U251 cells in a dose‐dependent manner. The above findings showed that scutellarin can inhibit the proliferation and growth of glioma cells in vivo and in vitro. In order to find out the mechanism of scutellarin against glioma, the possible target of scutellarin was obtained by bioinformatics and network pharmacology methods. Our experimental results indicated that scutellarin may to exert its anti‐glioma effect through BIRC5.

BIRC5 is a newly discovered member of the IAP family and has the strongest function among the inhibitors of apoptosis found so far.^14^ Apoptosis is a type of programmed cell death, and evading the regulation of the apoptotic cell death mechanism is an important tumour characteristic.^31^ A large amount of evidence showed that changes in apoptosis are not only related to the tumour occurrence and development, but also affect tumour resistance to drug therapy.^32^, ^33^, ^34^ Of note, it has been reported that glioma cells have blocked apoptosis and uncontrolled proliferation.^35^ However, the mechanism of tumour cell apoptosis escape is very complex, so understanding it and developing new drugs to improve the treatment of glioma is urgent. In the present study, it was found that BIRC5 expression was abnormally increased in U251 and LN229 cells, and scutellarin could significantly attenuate these changes. As compared with HEB normal glial cells, scutellarin also significantly decreased the expression level of BIRC5 in glioma cells.

In addition, we also studied whether scutellarin inhibits the proliferation of glioma cells through apoptosis, because the inhibition of apoptosis is an important factor in the uncontrolled proliferation of glioma cells.^36^ These results showed that scutellarin significantly promoted cell apoptosis, while the proliferation of glioma cells was significantly weakened.

In conclusion, the present study confirmed that scutellarin can inhibit glioma growth, as well as the proliferation and migration of glioma cells. In addition, scutellarin can significantly reduce BIRC5 expression in order to reverse glioma cell apoptosis inhibition and to exert its anti‐glioma effect. Therefore, scutellarin may become a new potential targeted drug for the treatment of glioma.

## AUTHOR CONTRIBUTIONS

**Feng Wang:** Project administration (equal); resources (equal); writing – original draft (equal). **Machicheng Bao:** Writing – review and editing (equal). **Jing Xu:** Writing – review and editing (equal). **Lanlan Shi:** Project administration (supporting). **Ruize Niu:** Project administration (supporting). **Hua Ting wang:** Project administration (supporting). **Jia Liu:** Project administration (equal); resources (equal).

## FUNDING INFORMATION

This work was supported by the Innovation Fundation of Yunnan Education Department (Project number: 2019Y0351) and Fundamental Research Project of Yunnan Province (Project number: 202201AS070082).

## CONFLICT OF INTEREST STATEMENT

The authors declare that there is no conflict of interest.

## CONSENT

Not applicable.

## Supporting information


**Appendix S1:** Supporting Information

## Data Availability

The datasets used and analyzed during the current study are available from the corresponding author on reasonable request.
