# Supplementary material for: Scutellarin inhibits the glioma cell proliferation by downregulating BIRC5 to promote cell apoptosis
Source: J Cell Mol Med. 2023 Jun 20;27(14):1975–87. doi: 10.1111/jcmm.17788 (PMC10339093; doi:10.1111/jcmm.17788)
Supplement: Supplementary file 1 — Appendix S1: Supporting Information [file JCMM-27-1975-s001.docx]

***Supplementary Material***

**Supplementary Results**

**1 The gene ID and differences of the 191 DEGs**

According to the transcriptome data of glioma in TCGA database, we analyzed and screened the glioma transcriptome and obtained 191 genes with significant differences. The gene ID and differences of these 191 genes were shown in supplementary table 1.

**Supplementary Table 1. 191 DEGs in glioma tissues according to TCGA**

| **Gene Symbol** | **Log FC** | **Gene Symbol** | **Log FC** | **Gene Symbol** | **Log FC** | |
| --- | --- | --- | --- | --- | --- | --- |
| KLK3 | -7.33628 | HOXB7 | 7.862444 | PI3 | 8.079496 | |
| SLC22A8 | -6.69999 | HOXA7 | 8.860624 | LINC02783 | 6.23688 | |
| NEUROD6 | -6.05737 | CHCHD2P8 | 7.228369 | C6orf15 | 10.86723 | |
| SNRPGP10 | 6.156867 | DPEP1 | 6.700516 | TFAP2B | 7.740406 | |
| H3F3AP6 | 6.3496 | HOXD8 | 8.329323 | LINC01385 | 6.142412 | |
| LINC01007 | -6.78647 | PITX1 | 7.980392 | TBX5 | 8.080188 | |
| RRM2 | 7.225175 | HOXC6 | 6.765754 | TUBBP6 | 6.442535 | |
| POTEF | 6.71305 | AC015911.1 | 6.126738 | PLA2G2A | 7.103753 | |
| LINC01476 | -6.16129 | HOXB3 | 7.228034 | TLX1 | 6.951432 | |
| KIF20A | 6.628297 | HIST1H2BH | 6.034149 | HOXC13-AS | 8.421223 | |
| DLGAP5 | 7.074048 | HOXA2 | 9.208823 | HAND2-AS1 | 6.357994 | |
| AC007922.3 | -6.3631 | NKX3-2 | 6.63427 | SAA2 | 7.346889 | |
| UBE2C | 7.272536 | MMP9 | 7.268662 | LBX1 | 6.320065 | |
| NDC80 | 6.001623 | HOXA4 | 8.520795 | HOXB5 | 6.746753 | |
| PBK | 7.196656 | HOXD13 | 9.871103 | ADGRG7 | 9.168587 | |
| TOP2A | 7.209457 | OTP | 6.988469 | HOXB8 | 8.250546 | |
| AC079328.1 | 6.106701 | HIST2H2AA4 | 6.117963 | FP236383.3 | 6.857741 | |
| MELK | 6.826454 | HOXA11 | 9.474 | FP671120.6 | 7.35482 | |
| E2F8 | 6.51348 | AC012005.1 | 6.071746 | MNX1-AS1 | 7.152785 | |
| MYBL2 | 7.614503 | HOXC-AS2 | 8.35637 | CAMP | 8.074376 | |
| AURKB | 6.579621 | LINC01587 | 6.306861 | HOXB13 | 6.331906 | |
| TROAP | 6.330061 | HOXC9 | 7.67658 | SIX6 | 8.553064 | |
| SHOX2 | 8.546588 | HOXC11 | 10.11747 | ELDR | 10.5501 | |
| BIRC5 | 6.238864 | MEOX2 | 6.005699 | KRT17P7 | 6.138768 | |
| AC098591.3 | 6.985225 | HIST2H2AA3 | 6.210479 | MMP8 | 7.099459 | |
| AL049830.1 | 7.401711 | HIST1H3G | 7.783146 | FGFBP2 | 6.050662 | |
| AC078991.1 | 7.623291 | HOXD4 | 6.625995 | IGF2BP1 | 6.68201 | |
| AC034207.1 | 6.880734 | LINC01956 | 6.302363 | CAPN6 | 9.039799 | |
| AC004057.1 | 6.360179 | HP | 9.215209 | PITX2 | 7.278003 | |
| PIMREG | 6.024067 | HOXB4 | 8.467553 | SEPTIN14 | 9.8836 | |
| H2AFZP2 | 6.621619 | HOXC8 | 7.12591 | CCDC140 | 6.298957 | |
| RPS11P7 | 6.858897 | FOXD3 | 6.464625 | TMEM270 | 6.19847 | |
| HOXA5 | 9.112397 | HOXB2 | 6.365578 | RNA5-8SP6 | 7.223625 | |
| SNRPD2P1 | 6.72245 | AC093895.1 | 7.507753 | HOXB-AS2 | 6.047139 | |
| FOXD3-AS1 | 7.557749 | HOTAIR | 8.969518 | CXCL13 | 7.202987 | |
| AL133163.1 | 6.451561 | LINC01571 | 6.055347 | TBX5-AS1 | 6.464901 | |
| LINC02732 | 9.850045 | BARHL1 | 6.090062 | MS4A6E | 6.483187 | |
| GSC | 7.064577 | CHI3L1 | 6.1089 | SIX3-AS1 | 6.391597 | |
| HOXD-AS2 | 9.592609 | EVX2 | 6.02779 | SAA2-SAA4 | 8.207448 | |
| IGF2BP3 | 6.181057 | HOXC13 | 8.820182 | MMP13 | 8.835759 | |
| SNRPFP1 | 6.178454 | HOXC-AS1 | 8.448827 | PRDM13 | 6.053732 | |
| HOXA1 | 6.884074 | HAND2 | 7.324814 | ORM1 | 6.876116 | |
| AL359704.1 | 6.774682 | HOXA11-AS | 7.160073 | MIR217HG | 6.496249 | |
| BTF3P4 | 6.163799 | HOXA9 | 8.36332 | GUCA2A | 6.560712 | |
| AC093155.2 | 6.010895 | AC020905.1 | 6.107188 | HIST1H3F | 6.541177 | |
| SRIP3 | 6.369323 | HOXC-AS3 | 6.869984 | AC074351.1 | 6.519859 | |
| HOXA3 | 9.141008 | IL21-AS1 | 6.752404 | IGKV1-9 | 8.183461 | |
| HOXD10 | 11.44066 | LTF | 8.469088 | LINC01765 | 6.497265 | |
| AL590135.1 | 6.737933 | SCNN1B | 6.614348 | CCL18 | 7.218003 | |
| AC011933.1 | 6.094052 | HOXC5 | 6.101837 | ELSPBP1 | 6.137613 | |
| RPL10P5 | 6.172252 | POSTN | 7.313023 | IGLV2-14 | 6.039498 | |
| AC010904.1 | 6.037129 | HOXB6 | 6.673841 | PRAC2 | 6.045596 | |
| HOXA-AS3 | 8.868831 | LINC01198 | 6.586089 | DEFA3 | 6.699969 | |
| HOXA10 | 9.035805 | COL3A1 | 6.174806 | HOXB-AS3 | 6.688309 | |
| HOXD9 | 9.724476 | GATA4 | 8.284146 | FOXA2 | 7.177269 | |
| NKX2-5 | 9.955282 | CA3 | 6.238097 | C5orf46 | 6.854409 | |
| EN1 | 8.340419 | HOXA13 | 6.337532 | WT1-AS | 6.647409 | |
| HOXA6 | 8.81484 | SAA1 | 8.879186 | IGHV4-39 | 6.654437 | |
| ADAMDEC1 | 9.41459 | AL606970.4 | 7.07563 | IGLV3-1 | 8.067897 | |
| Z99714.1 | 6.17615 | MNX1 | 7.237619 | STOML3 | 6.27837 | |
| HOXA-AS2 | 8.533502 | PRAME | 10.14652 | LINC01602 | 7.854514 | |
| HOXD11 | 8.914598 | IDO1 | 6.23839 | AL139231.1 | 7.099673 | |
| LINC02308 | 6.162103 | HOXB9 | 6.891923 | MAGEC2 | 6.708891 | |
| HOXC10 | 9.369177 | H19 | 7.281864 |  |  |  |

**2 Effect of scutellarin on LN229 and HEB cells.**


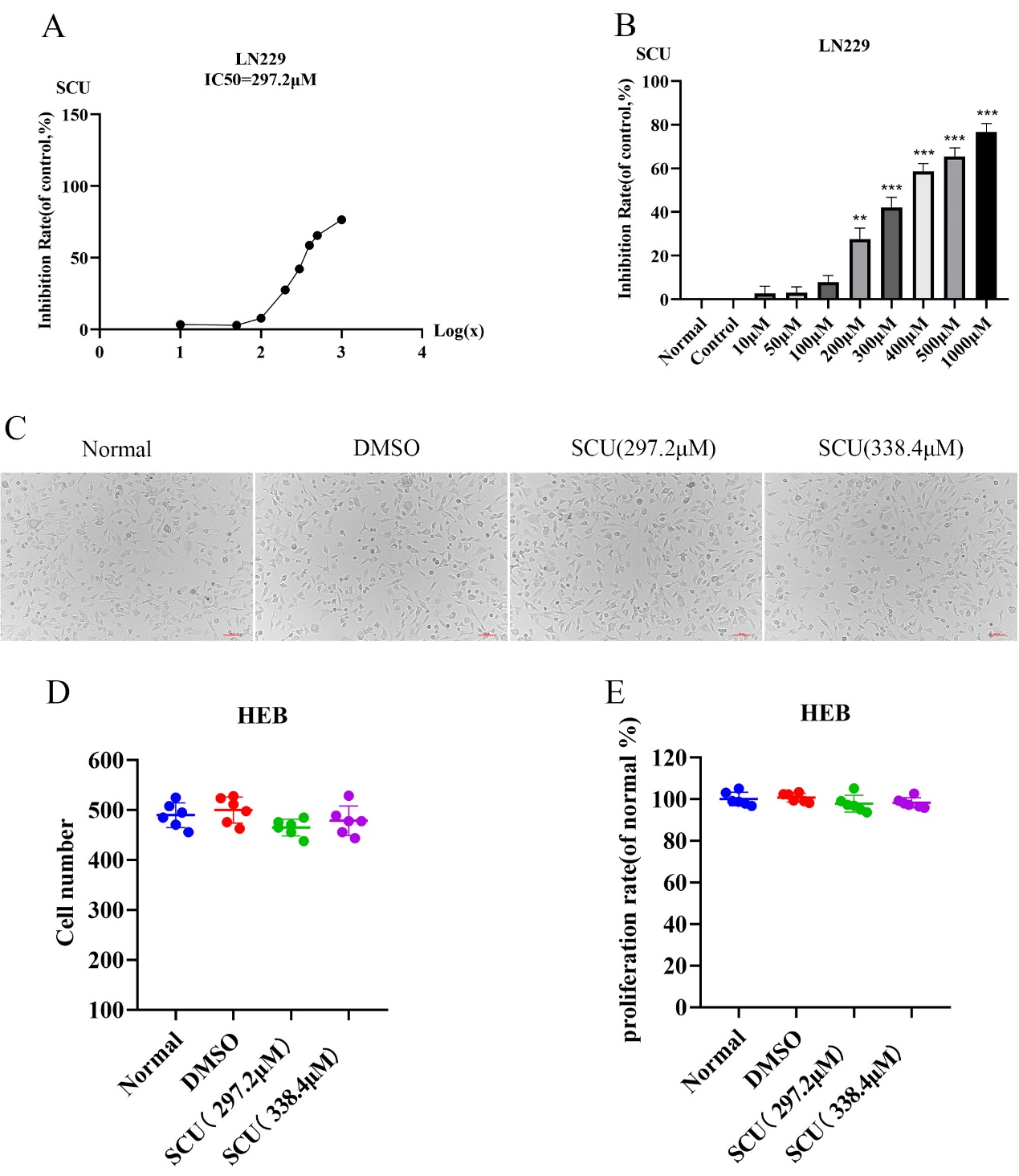


Supplementary Figure 1: Effect of scutellarin on LN229 and HEB cells. (a) IC50 of scutellarin in LN229 cells. (B) The inhibitory effect of scutellarin on the proliferation of LN229 cells (n=6). (C) Morphological changes of HEB cells at the concentrations of 297.2 and 338.4 μM. (D) Number of HEB cells at the concentrations of 297.2 and 338.4 μM (n=6). (E) Effect of 297.2 and 338.4 μM on the proliferation of HEB cells (n=6). Data are expressed as mean ± standard deviation, **0.01<p<0.05，***p<0.001.

**3 Effect of scutellarin on the migration of U251 cells.**


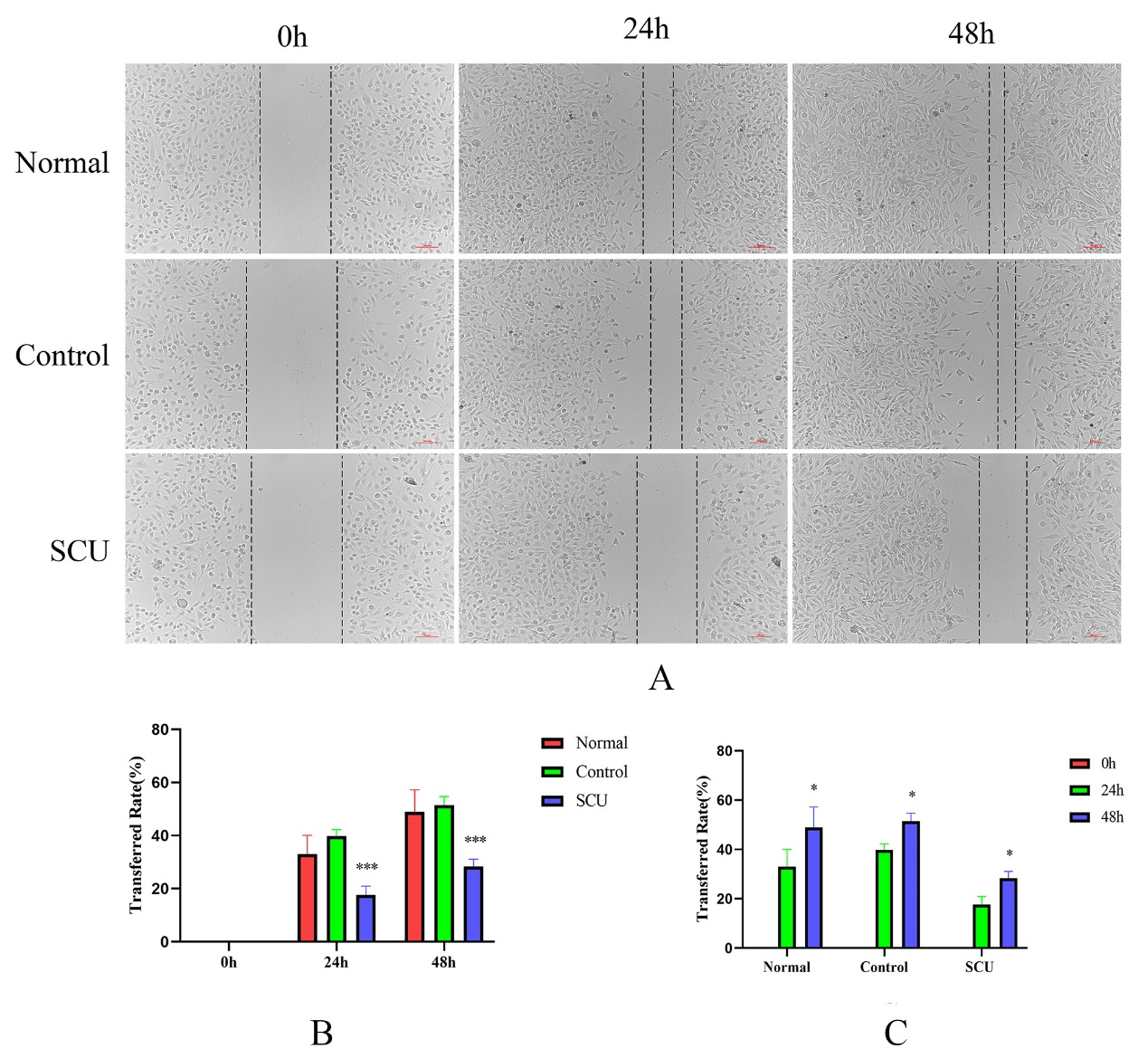


Supplementary Figure 2: Effect of scutellarin on the migration of U251 cells. (A) U251 cell scratch test. (B, C) In the scratch experiment, the cell migration rate after adding scutellarin at 0h, 24h and 48h(n=3). Data are expressed as mean ± standard deviation, ***p<0.001, *p<0.05.

**4 Identification of samples according to clinical glioma patients**


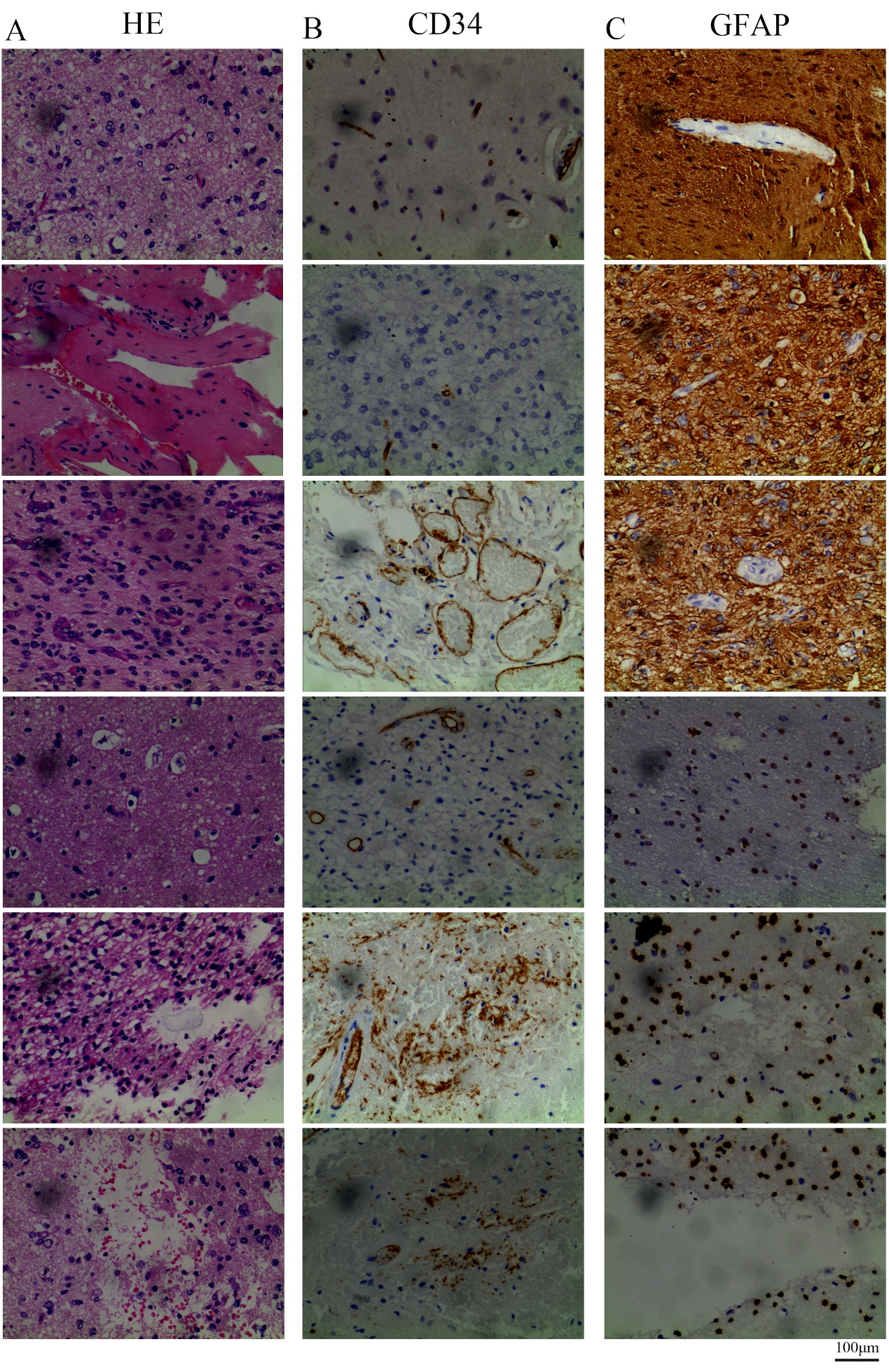


Supplementary Figure 3: Identification of glioma tissue samples. (A) Paraffin sections of glioma tissue were stained with HE. 200× (n=6). (B) Immunohistochemical staining (CD45+) of paraffin sections of glioma tissues. 200× (n=6). (C) Immunohistochemical staining (GFAP+) of paraffin sections of glioma tissues. 200× (n=6).

**5 Screening of BIRC5 interference fragments and the effect of BIRC5 knockdown on glioma cells**


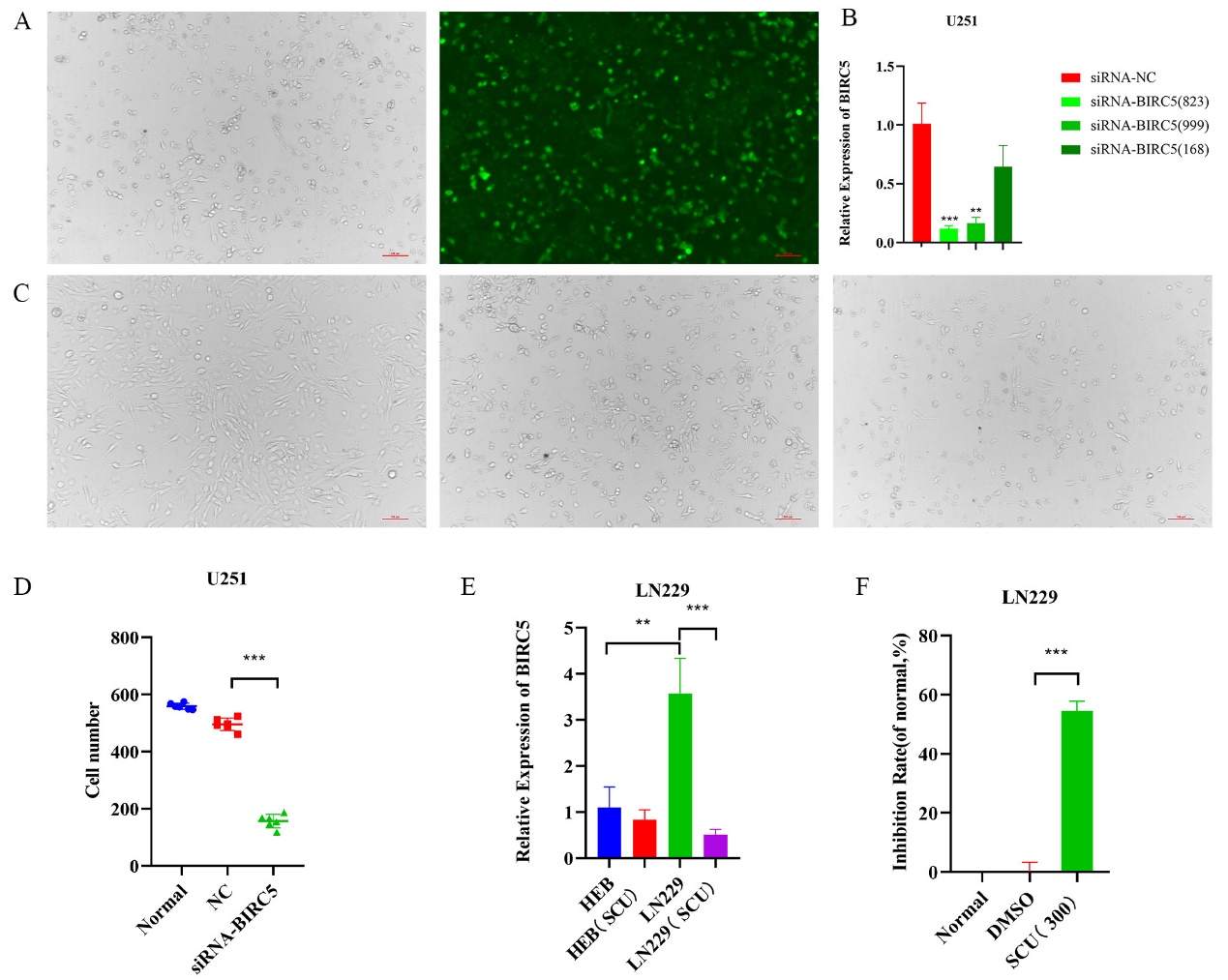


Supplementary Figure 4: Screening of BIRC5 interference fragments and the effect of BIRC5 knockdown on glioma cells. (A) Identification of BIRC5 transfection efficiency. (B) Screening of BIRC5 transfection efficiency (n=3). (C) The morphological changes of U251 cells after BIRC5 knockdown were observed, from left to right are Normal, DMSO (0.2%) and siRNA-BIRC5. (D) The effect of BIRC5 knockdown on the number of U251 cells (n=6). (E) The relative expression of BIRC5 in HEB and LN229 cells treated with scutellarin for 48 h (n=4). (F) The inhibition rate of scutellarin (300 μM) on LN229 cells after 48h treatment (n=6). Data are expressed as mean ± standard deviation, *p<0.05，**0.001<p<0.01，***p<0.001.
